# A nurse- and peer-led support program to assist women in gynaecological oncology receiving curative radiotherapy, the PeNTAGOn study (Peer and nurse support trial to assist women in gynaecological oncology): study protocol for a randomised controlled trial

**DOI:** 10.1186/1745-6215-14-39

**Published:** 2013-02-11

**Authors:** Penelope Schofield, Ilona Juraskova, Rebecca Bergin, Karla Gough, Linda Mileshkin, Meinir Krishnasamy, Kate White, David Bernshaw, Sylvia Penberthy, Sanchia Aranda

**Affiliations:** 1 Department of Cancer Experiences Research, Peter MacCallum Cancer Centre, 3002, East Melbourne, Vic, Australia; 2 Sir Peter MacCallum Department of Oncology, The University of Melbourne, 3010, Parkville, Vic, Australia; 3 School of Health Sciences, Department of Nursing, The University of Melbourne, 3010, Parkville, Vic, Australia; 4 Centre for Medical Psychology & Evidence-based Decision making, University of Sydney, 2006, Sydney, NSW, Australia; 5 Department of Gynaecological Oncology, Peter MacCallum Cancer Centre, 3002, East Melbourne, Vic, Australia; 6 Sydney Nursing School, University of Sydney, 2006, Sydney, NSW, Australia; 7 Sydney Cancer Centre, Royal Prince Alfred Hospital, 2050, Camperdown, NSW, Australia; 8 Cancer Institute NSW, PO Box 41, 1435, Alexandria, NSW, Australia

**Keywords:** Peer support, Nurse, Intervention, RCT, Gynaecological cancer, Radiotherapy, Distress, Quality of life, Psychosexual function

## Abstract

**Background:**

Women who undergo radiotherapy for gynaecological cancer (GC) can experience distressing side effects which impact on psychosocial functioning and intimate relationships. Cancer-related distress may be ameliorated by comprehensive preparation for treatment and addressing women’s informational, physical, psychological and psychosexual needs. This paper describes the protocol for a multisite randomised controlled trial (RCT) testing a novel intervention package which combines tailored specialist nursing consultations and telephone peer support with the primary aim to reduce psychological distress. Secondary aims assess patient quality of life, symptom distress, unmet supportive care needs, preparation for treatment, psychosexual functioning and vaginal stenosis.

**Methods/design:**

This multifaceted intervention comprises four nurse-led consultations coupled with four phone calls from a peer support volunteer (GC survivor). The evidence-based intervention will be delivered at critical points in the illness trajectory: pre-treatment, mid-treatment, treatment completion and post-treatment. Nurses and peers undergo 2-day intensive training workshops, are guided by comprehensive study intervention manuals and receive ongoing supervision and support. Eligible patients will have a diagnosis of GC, be scheduled to receive curative radiotherapy, be aged 18 years or over and speak English. Three-hundred and six participants will be randomized to receive usual care or usual care with the intervention package. Study outcome measures will be collected at baseline, day 1 of radiotherapy and 1, 6 and 12 months post radiotherapy. Clinical assessments of vaginal toxicity will occur at baseline, and 3, 6, and 12 months post radiotherapy.

**Discussion:**

This timely research has the potential to substantially reduce the physical, psychosexual and supportive care needs of women with GC. Using a telephone peer support model, the intervention package ensures equitable access to support services for geographically isolated patients. The novel intervention engages peer volunteers who liaise with nurses to encourage adherence to professionally-delivered information and provide emotional support. It has been designed to be potentially transferable to a range of treatment settings and diseases. Based on pilot data, the proposed intervention was found to be useful and acceptable to patients and clinicians. If effective and feasible in the multisite RCT, the program could be widely disseminated.

**Trial registration:**

Australian New Zealand Clinical Trial Registry ACTRN12611000744954

## Background

### Physical and psychological burden of disease

Gynaecological cancers (GC) account for 19% of all female cancers worldwide [1], and in addition to the psychological implications of living with cancer, women with GC experience significant psychosexual and psychosocial issues unique to their diagnosis [2,3]. A common treatment for GC is radiotherapy, which can cause many distressing side effects that have both immediate and late impacts on quality of life (QoL) [4]. These side effects include diarrhoea [5], abdominal cramps [5], bladder dysfunction [6], menopause, infertility [6] and sexual dysfunction [6]. Women may also experience vaginal side effects including stenosis, atrophy, agglutination, reduced genital sensation, vaginal dryness, dyspareunia and postcoital bleeding [2,7]. As these side effects involve intimate and private aspects of bodily function their presence also has a major impact on close relationships and social functioning.

Not surprisingly, upwards of 40% of GC patients report chronic and distressing sexual difficulties [2], which may continue over the 12 months post treatment [8]. GC diagnosis also commonly impacts upon a woman’s self-esteem, body image, femininity and intimate relationships [2,3]. Yet up to half of GC patients report not having any discussions with their clinicians about post-treatment sexual adjustment [8,9], which represents a concerning gap in the provision of healthcare.

In addition to the physical impact, over half of patients with cancer feel anxious prior to treatment and about 40% remain anxious at treatment completion [3]. High anxiety has been associated with poorer sexual functioning [8], worse QoL up to 1 year post treatment [10] and lower social support among women with GC [11]. However, anxiety levels may be ameliorated by both comprehensive preparation for treatment and addressing needs during treatment [12,13].

A Cochrane review published in 2008 recommends women use a vaginal dilator and/or engage in regular sexual intercourse [7] to minimise vaginal stenosis and agglutination after pelvic radiotherapy for GC. Patient adherence to such strategies is associated with less physical damage and greater sexual satisfaction [7,14], and allows adequate pelvic examination to detect cancer recurrence [15]. However, adherence is suboptimal [7,8]. Moreover, a more recent Cochrane review generated debate over the appropriate use of dilators by concluding that there was insufficient data to recommend routine use [16]. While recommended practice varies across treatment centres [15], current practice guidelines internationally advocate the use of a vaginal dilator for women receiving pelvic radiotherapy [17-20].

### Innovative, evidence-based model of supportive care

Given the physical and psychological impact of GC, there is a high need to provide appropriate and timely interventions to women with these cancers. An evidence-based intervention combining nurse-led consultations with peer telephone support has the potential to address these needs.

Level 1 evidence shows that providing sensory and procedural information, and addressing patients’ fears about surgery results in less pain, distress and fewer days in hospital [12,13]. Only two studies have applied these principles to the radiotherapy context; one found provision of sensory and procedural information about radiotherapy resulted in reduced anxiety [21]; the other, a RCT involving taped procedural and coping information, resulted in increased perceived knowledge and self-efficacy [22]. This high level evidence has been incorporated into the study design as a radiotherapy area tour.

A systematic review showed that there are effective, evidence-based self-care strategies for side effects common to several cancer treatments such as fatigue, diarrhoea and infertility [23]. In addition, involving patients with chronic disease in their disease management results in improved self-reported health and lower psychological distress, less health system use and reduced health costs [24,25]. It is therefore likely that women treated for GC with radiotherapy would benefit from a coordinated provision of individualised, evidence-based interventions and resources to optimise their recovery.

Nurse-led consultations and telephone interventions for cancer patients have been shown to be appropriate and effective [26]. Of interest, health professional delivery of tailored information to women with GC about side effects and effective self-care has been linked to better coping with side effects; compliance with post-radiation rehabilitation; less fear about sexual intercourse and less relationship disruption [14,27]. In addition, evidence from a RCT shows that nurse coordinated multidisciplinary and community referrals result in better symptom control for cancer patients [28]. Aranda and colleagues have recently demonstrated that a nurse-led pre-chemotherapy educational consultation was effective in reducing pre-treatment anxiety amongst those experiencing moderate to high anxiety at baseline [29]. As such, individualised and targeted nursing consultations with provision of evidence-based self-care strategies are an integral component of the intervention design.

Multidisciplinary care (MDC) refers to a team approach to healthcare delivery that involves input from all relevant medical, nursing and allied health areas. MDC has been linked to a number of improvements in disease outcomes, including survival benefits and better QoL, lower distress, decreased length of hospital stay, reduction in healthcare costs, improved staff satisfaction and improved knowledge of patient care [3,27,30]. No studies testing a system to facilitate MDC for women with GC have been identified. Effective referral requires that clinicians have excellent communication skills to elicit and respond to patient emotional and information cues. A systematic review showed that communication skills training programs improve clinicians’ skills and confidence in psychological assessment and interviewing [31]. The intervention includes training of study nurses to facilitate appropriate, timely and effective MDC referrals.

In addition to nursing interventions, models of psychosocial cancer care have identified a pivotal role for peer-support programs [32]. The unique perspective of a peer facilitates sharing and practical, social and emotional coping [33,34]. A systematic review of cancer peer-support programs indicated high satisfaction and perceived psychosocial benefits among participants [33]. Some patients even prefer peer-delivered over professionally-delivered support [35]. Telephone models of peer support have additional economic and logistic advantages, and are successful in reaching housebound or geographically isolated patients [36].

Despite widespread use and positive participant perceptions [33], only two RCTs testing one-to-one telephone peer support have been identified in the cancer literature [37,38]. Rudy and colleagues found that perceived social support was higher among melanoma patients receiving telephone peer support [38]. The second study reported no significant difference in newly diagnosed GC patient’s emotional distress with peer telephone support, however the sample size was small (*n*=32) [37]. No RCTs investigating peer-support interventions from treatment initiation to post-treatment completion were identified [33,35], and none have been found that combine tailored nurse consultations with telephone peer support.

This study will evaluate the effect of an innovative, nurse-led intervention combined with telephone-based peer support to provide evidence-based information and resources, coaching in self-care, multidisciplinary referrals and psychological support to optimise the recovery of women treated for GC with radiotherapy.

### Study aims

The primary aim is to evaluate the effectiveness of the intervention package to reduce psychological distress for women receiving radiotherapy with curative intent for GC. Secondary aims are to examine the impact of the intervention on patient quality of life, symptom distress, unmet supportive care needs, preparation for treatment, psychosexual functioning and vaginal stenosis.

### Study hypotheses

#### Primary endpoint

Compared to the usual care group, the intervention group will report lower psychological distress from baseline to first follow-up immediately prior to the first radiotherapy treatment, and follow-up at 4 weeks post treatment.

#### Secondary endpoints

Compared to the usual care group, the intervention group will report:

1. Lower informational and psychosocial supportive care needs and lower symptom distress from baseline to follow-up at 4 weeks post treatment,

2. Better preparation for treatment from first follow-up immediately prior to the first radiotherapy treatment to follow-up at 4 weeks post treatment,

3. Higher psychosexual functioning from baseline to follow-up at 6 and 12 months post treatment,

4. Higher quality of life from baseline to follow-up at 4 weeks post treatment, and

5. Less vaginal stenosis at 3, 6 and 12 months post treatment.

## Methods

### Design and setting

The trial takes place in six sites from three states of Australia. All sites are public hospitals, with two sites part of a specialist oncology facility. The volume of patients through each centre differs, so sites will not contribute equally to participant recruitment.

Ethical approval has been obtained from the Human Research Ethics Committees of participating states (Peter MacCallum Cancer Centre Ethics Committee, Project No: 09/07; Ethics Review Committee Royal Prince Alfred Zone, Project No: X11-0112 & HREC/11/RPAH/154; Royal Brisbane & Women’s Hospital Human Research Ethics Committee, Ref No: HREC/11/QRBW/202).

This study is a multicentre, prospective, randomised controlled intervention trial for patients receiving radiotherapy for gynaecological cancer, with follow-up of 1 year post-end of treatment.

### Participants

Patients will be consecutively screened for eligibility at participating sites. Inclusion criteria are: (1) have a confirmed diagnosis of gynaecological cancer; (2) be scheduled to receive radiotherapy with curative intent to the pelvis; (3) be aged 18 years or older; and (4) be able to read and write English, and give informed consent. Exclusion criteria are: (1) a severe psychiatric or cognitive disorder; (2) treatment with palliative intent; or (3) previous treatment with radiotherapy to any part of the body.

Patient eligibility will be confirmed by the treating clinician and the trained data manager will provide additional information and gain written informed consent. Prior to radiotherapy commencement, the treating clinician will complete a vaginal examination and document baseline vaginal toxicity. The data manager will be responsible for liaising with the trained nurses and peers to organise and coordinate intervention delivery.

### Intervention

**Figure 1:**
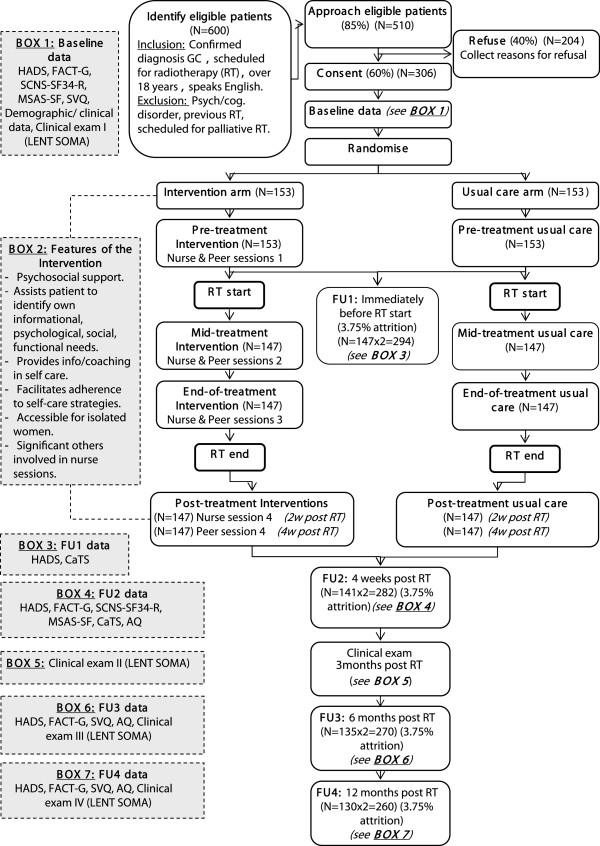
**PeNTAGOn study design: a randomised controlled trial.** Schematic diagram of the PeNTAGOn study design. Patients will be screened for eligibility (criteria listed), then approached and randomised to receive usual care or usual care with the nurse and telephone peer support intervention. Critical intervention time-points are highlighted pre-, mid-, end- and post-treatment. Time-points for follow-up data collection to 12 months post end of radiotherapy are noted. Estimates for sample recruitment and retention are shown in each step of the diagram. Box 1 describes baseline data collected. Box 2 describes key features of the intervention. Boxes 3 to 7 describe follow-up data collection. AQ, Adherence questionnaire; CaTS, Cancer Treatment Survey; FACT-G, Functional Assessment for Cancer Therapy - General; GC, Gynaecological cancer; HADS, Hospital Anxiety and Depression Scale; LENT SOMA, Late Effects of Normal Tissues / Subjective-Objective Management Analytic; MSAS-SF, Memorial Symptom Assessment Scale Short Form; RT, Radiotherapy; SCNS-SF, Supportive Care Needs Survey - Short Form; SVQ, Sexual function-vaginal changes questionnaire.

The psychosocial intervention will involve two linked components: (1) nurse-led consultations; and (2) peer telephone support. These elements will be delivered at four critical points in the illness trajectory: pre-treatment, mid-treatment, treatment completion and post-treatment. Figure 1 provides a schematic diagram of the intervention components.

#### Nurse-led consultations

##### Training

Two specialist cancer nurses will be trained to provide the consultations at each site. The intervention nurse will not be involved in the management of ‘usual care’ patients in order to prevent diffusion of the intervention. Training will comprise online education in distress management, aspects of survivorship and modules on the psychosexual care of women with GC. Training also includes a 2-day workshop facilitated by experts in communication, cancer nursing, patient education and coaching, and psychosexual rehabilitation. The workshop learning objectives are to: (1) gain an understanding of the project including preventing intervention diffusion; (2) develop skills to elicit, explore and respond to patient concerns; and (3) coach and communicate effectively with patients on the use of evidence-based self-care strategies and psychosexual issues. Specific strategies include pelvic floor exercises and the use of vaginal dilators and moisturisers. Training methods included didactic and interactive teaching, rehearsal of skills and constructive feedback using role-plays with actor-patients and the provision of a detailed intervention manual.

##### Intervention session 1: pre-treatment, face-to-face, 1 hour

Patients with cancer often report increased unmet needs related to the worries and fears of family members [39], so women will be encouraged to bring a significant other to the consultation. Given that anxiety related to treatment is typically highest just prior to starting treatment [3] and based on Level I evidence on treatment preparation [12,13] each patient will be given a brief tour of the treatment unit followed by a private consultation. The nurse will first ascertain the patient’s understanding of their situation and clarify misconceptions. A radiotherapy question prompt sheet developed from the literature and with consumer and professional input will be administered by the nurse and at the first three sessions to guide tailoring of the intervention. The patient identifies her top three concerns for focus in the session, with further concerns addressed at future sessions. The prompt sheet also assesses global distress using the Distress Thermometer [40]. Anxious patients will be trained to use the strategy of controlled breathing with positive self-talk, an effective self-care activity to reduce treatment-related anxiety [41]. The prompt sheet will aid a discussion of the woman’s supportive care needs and possible MDC referrals. The nurse will discuss vaginal health, psychosexual rehabilitation, and if appropriate, menopause and infertility. Coaching and rehearsal of self-care for side effects and stress-reduction will be offered, and tailored fact-sheets and information provided which can improve recall [30]. At the end of each session the nurse arranges a time for a call with the peer volunteer. The nurse also confirms any particular concerns the patient does not wish to discuss with her peer.

##### Intervention session 2: mid-treatment, face-to-face, 30 minutes

Since side effects of radiotherapy commonly commence at 2 to 4 weeks into treatment [17], this session addresses side effects and coaching in self-care strategies. The woman will be provided with lubricants, informed about and shown how to use the dilator and perform pelvic floor exercises. The timing of commencing dilator use will be according to local hospital policy. In addition, the nurse will assess the patient’s experience of treatment, normalise fears and ask about the call with her peer. The nurse will address any ongoing issues from the first session and new concerns and provide coaching in relevant self-care. Barriers to self-care and stress-reduction strategies will be elicited and the importance of adherence reinforced [42].

##### Intervention session 3: end of treatment, face-to-face, 30 minutes

Some patients report relief upon completing treatment however research by Jefford *et al*. has shown that this is often a time of uncertainty and anxiety [43]. This may be compounded by treatment side effects, both acute and long-term [2,5,6,14]. In this session the woman can discuss anxieties related to treatment completion, ongoing issues and treatment side effects with the nurse. Vaginal health and psychosexual recovery will be explored, including the resumption of sexual activity if desired. The nurse will provide the patient with a Survivorship Care Plan (SCP) detailing the woman’s diagnosis and treatment received, planned follow-up schedule, common ongoing or new treatment side effects, and how to manage them. With the patient’s permission, the nurse will fax a copy of the SCP to the patient’s primary care doctor.

##### Intervention session 4: 2 weeks post-treatment, telephone, 30 minutes

Many people report feeling abandoned and isolated after leaving the hospital system and an expectation by family and friends to return to ‘normal’ [43]. The nurse will explore concerns and experiences since completing treatment, address barriers to using self-care strategies and reinforce the importance of vaginal dilator use. The nurse will elicit new or remaining concerns and respond accordingly before prompting for any final questions.

#### Telephone peer support

Each woman will be linked with a peer who will provide four telephone support sessions throughout the treatment course. Peers will be matched to the participant’s medical and personal circumstances such as diagnosis, treatment type, age and area of residence. Approximately 30 peers will be trained over the course of the trial, accounting for peers taking breaks or withdrawing from the study. Each peer will support a maximum of two patients at any one time.

##### Training

Careful selection and training of peers and ongoing professional supervision is critical for the success of peer support programs [34]. The shortcomings of previous peer support studies, particularly regarding inadequate reporting of peer recruitment, training and support, will be overcome through a rigorous multistage selection and training process, the provision of ongoing training, supervision and debriefing by an experienced cancer nurse, and provision of a detailed intervention manual [44]. Peers will be past GC patients identified by clinical staff at participating centres and sent an invitation letter about the program. Peers must be over 18 years old, speak English and be at least 2 years post treatment. Those with cancer recurrence or a history of major psychiatric illness are ineligible. Experts in assessing peer volunteers for phone-based cancer peer-support programs will conduct screening phone interviews with interested peers. Suitable women attend a 2-day workshop. The workshop learning objectives are to: (1) gain an understanding of the project including privacy and confidentiality; (2) develop skills in active listening and providing empathy; and (3) reinforce self-care strategies recommended by the nurse. In subsequent weeks, they complete practice calls with an actor-patient to apply these skills, and feedback is provided by a communication skills expert.

##### Peer intervention sessions

The peer will contact the patient 1 week after each nurse consultation except for the last session when the peer calls the patient 4 weeks post treatment. The nurse will call the peer after their consultation with the patient, and send a check-list of the patient’s concerns and the individualised self-care plan. Using a structured format, the peer’s role will be to:

(1) Provide psychosocial support to the patient: Using open questions, empathy and active listening, the peer will establish rapport with the patient, hear her story and experiences and normalize the woman’s emotional reaction.

(2) Encourage adherence to the recommended self-care strategies: The peer will be trained to reinforce information and coaching on basic self-care strategies recommended by the nurse, including problem solving difficulties. For more complex problems or additional needs, the peer will encourage the patient to contact the nurse or access additional sources of information and support, such as the Cancer Council Helpline. If the peer has concerns about the patient, she will contact the nurse and discuss them.

### Sample size

The treatment effect is conservatively estimated to result in group differences of 0.35 standard deviations for continuous outcome measures [45-47]. With the Hospital Anxiety and Depression Scale total score (HADS-T) as the primary outcome, to achieve ≥80% power at a 5% significance level, the required sample size in each arm is 130 at the 12-month follow-up. With a patient approach rate estimated at 85%, consent rate estimated at 60% and attrition estimated at 15%, 600 participants will need to be approached to achieve a sample of 260.

### Study integrity

The study design and reporting will adhere to the Consolidated Standards of Reporting Trials (CONSORT) statement [48]. Usual care practice will be monitored yearly with a questionnaire administered to nurses most involved with the usual care of women with GC receiving radiotherapy at each site. A consecutive sample will be randomly allocated at the central site by computer with a weighted-biased coin method 1:1 to either intervention or usual care arms. Participants will be stratified according to treating hospital and treatment type. Minimisation will be used to balance the randomisation across the strata.

Participants will not be informed of their allocation until the baseline questionnaire has been received. Blinding of patients and providers to experimental arm cannot be achieved with this study design, however outcome assessment will be by self-reported questionnaire, thereby obviating the need for researcher blinding. Usual care arm participants will receive a booklet on their specific cancer diagnosis, treatment, and psychosexual recovery to meet minimal ethical standards [49], and information will be provided as per usual practice by their treatment team. Reasons for attrition will be recorded and recruitment and dropout bias assessed.

All nurse and peer sessions will be audio-taped and a random sample of 15% of sessions will be assessed for adherence to protocol. Length of sessions will be reported. The first five calls or consultations made by each peer and nurse will be reviewed and feedback provided on adherence to protocol, use of communication and coaching skills and areas for improvement.

### Measures

Patients will complete self-report, pen and paper measures. Clinical vaginal assessments will be completed by the treating clinician. Table 1 presents when measures will be administered.

**Table 1:** Time-points for collection of patient reported outcomes and clinical outcomes for the PeNTAGOn study

**Time-point**	**Questionnaire**
Baseline & clinical exam I: (pre-treatment)	Demographic and clinical variables, HADS, FACT-G, SCNS-SF34-R, MSAS-SF, SVQ, clinical exam I (LENT SOMA scale)
Follow-up 1: (immediately prior to first radiotherapy)	HADS, CaTS
Follow-up 2: (4 weeks post radiotherapy and post intervention)	HADS, FACT-G, SCNS-SF34-R, MSAS-SF, AQ, CaTS and Patient Care evaluation & Referrals
Clinical exam II (3 months post radiotherapy)	Clinical exam II (LENT SOMA scale)
Follow-up 3 & clinical exam III: (6 months post radiotherapy)	HADS, FACT-G, AQ, SVQ, clinical exam III (LENT SOMA scale)
Follow-up 4 & clinical exam IV: (12 months post radiotherapy)	HADS, FACT-G, AQ, SVQ, clinical exam IV (LENT SOMA scale)

AQ, Adherence questionnaire; CaTS, Cancer Treatment Survey; FACT-G, Functional Assessment for Cancer Therapy - General; HADS, Hospital Anxiety and Depression Scale; LENT SOMA, Late Effects of Normal Tissues / Subjective-Objective Management Analytic; MSAS-SF, Memorial Symptom Assessment Scale Short Form; SCNS-SF34-R, Supportive Care Needs Survey - short form, Revised response format; SVQ, Sexual function-vaginal changes questionnaire.

#### Demographics and clinical variables

Demographic details will be recorded by the patient on the baseline questionnaire and include age, postcode, marital and education status, employment situation and occupation, living arrangements, whether they have children, sexual orientation, country of birth and first language, menopausal status, and antidepressant or sedative use. Clinical details of diagnosis, diagnosis date, disease stage, Eastern Cooperative Oncology Group (ECOG) performance status, histopathology grade, history of another cancer, treatment for a past diagnosis, current treatment type (external beam, brachytherapy, concurrent chemotherapy), prior treatments (surgery, chemotherapy), involvement with hospital services and Charlson co-morbidity index will be completed by the data manager on a case record form.

#### Clinical examination

The treating clinician will conduct clinical vaginal examinations. The LENT SOMA scale (Late Effects of Normal Tissues / Subjective-Objective Management Analytic), objective criteria for vaginal/sexual dysfunction [50,51] will be used to measure vaginal changes.

#### Psychological distress

Psychological distress will be assessed with the 14-item HADS total scale (HADS-T) [52]. Rasch analysis shows that items comprising the HADS-T form a unidimensional construct of psychological distress [53]. It has demonstrated high internal consistency (alpha=0.82 to 0.90) in patient populations including cancer patients [54,55] and responsiveness in psychosocial intervention studies [56,57].

#### Cancer-specific quality of life

Cancer-specific quality of life will be assessed with the 27-item Functional Assessment for Cancer Therapy - General (FACT-G). The FACT-G comprises four subscales for specific quality of life domains: physical, social, emotional and functional wellbeing. Scaling and unidimensionality of subscales have been confirmed by factor and Rasch analyses [58,59]. All subscales have demonstrated high internal consistency (alpha=0.72 to 0.90), good convergent (r>0.51), divergent (r<0.22) and discriminative validity in cancer patients [45] and responsiveness in psychosocial intervention studies [57,60].

#### Unmet supportive care needs

The 34-item Supportive Care Needs Survey-short form with revised response format (SCNS-SF34-R) [61,62] covers unmet needs from five domains: psychological; health system and information; physical and daily living; patient care and support; and sexuality. All domain subscales have high internal consistency (alpha≥0.87) and good divergent and convergent validity [63]. Aranda, Schofield and colleagues found this scale to be responsive to change in a recent RCT with cancer patients [64].

#### Symptom distress

The 32-item Memorial Symptom Assessment Scale Short Form (MSAS-SF) [65] comprises three subscales (physical, psychological and global distress) and a total symptom distress scale. All scales have demonstrated high internal consistency (alpha=0.76 to 0.87), good convergent validity (r =−0.68 to −0.74), excellent discriminative validity based on known groups comparisons [65] and sensitivity to change in a longitudinal setting [66].

#### Sexual function and vaginal changes

The 27-item Sexual function-vaginal changes Questionnaire (SVQ) [67], developed for gynaecological cancer, comprises three scales for all patients (intimacy, sexual interest and global sexual satisfaction) and two scales for sexually active respondents (vaginal changes and sexual functioning). All five scales have demonstrated high internal consistency (alpha =0.76 to 0.83) [68] and sensitivity to change longitudinally [67].

#### Cancer treatment-related information and support needs

Schofield *et al*. developed a 25-item Cancer Treatment Survey (CaTS) [69] which comprises two subscales for specific information and support needs domains: sensory-psychological concerns and procedural concerns. Both subscales have demonstrated high internal consistency (alpha>0.90) and good divergent validity (with HADS: r<0.26) [69]. Both were sensitive to change in a recent RCT [29].

#### Adherence questionnaire and patient care evaluation

Adherence to use of vaginal dilator, moisturiser, lubricant and pelvic floor exercises will be assessed with a purpose designed Adherence questionnaire (AQ). Patients’ perceived global rating of change in psychological distress from baseline and experience of referrals from commencement of treatment will be assessed with a patient care evaluation questionnaire, also purpose designed.

### Statistical analyses

All data will be analyzed through SPSS Windows Version 20.0 (SPSS, Chicago, IL, USA). After inspection of the data, the appropriateness of all methods described below will be reviewed and revised if necessary.

#### Preliminary analysis

Descriptive statistics will be used to summarise baseline data, compliance with questionnaires and reasons for non-compliance by study arm. Parametric and non-parametric tests of association and mean differences as appropriate will be used to assess recruitment bias and possible differential attrition for consenting participants.

#### Outcome analysis

Outcome analyses will be carried out by fitting linear mixed models (LMM) to each outcome separately. In this case, a sequence of two-level models including random intercepts and slopes will be constructed for each outcome following recommended procedures [70]. Fully parameterised models will also include fixed effects for time (linear and higher-order polynomials, as appropriate), group, site plus all two-way and cross-level interactions. LMM use all available data, which supports an intention-to-treat approach, and adjusts variance estimates for the correlation between repeated measurements on the same participants. Potential confounders (that is, patient characteristics such as age) will be included as co-variates. These will be centred to facilitate accurate interpretation of results and will be retained in the final models if they explain significant variation in outcomes and improve the precision of the estimates of the treatment effect.

#### Secondary descriptive and planned subgroup analysis

For descriptive purposes, observed data will be used to calculate within- and between-groups changes in study outcomes from first assessment to follow-ups as specified in the study hypotheses. Effect sizes for between-groups differences will be calculated using recommended procedures [71]. Interpretation of changes in the primary outcome will be facilitated by results from an analysis of participants’ global ratings of change in psychological distress [72,73]. For the primary outcome, subgroup analysis will be used to investigate whether the intervention effect differs significantly between participants with and without clinically significant distress at baseline.

## Discussion

This study will examine the effectiveness of an innovative, tailored, nurse and peer-support package for women with GC. Drawing on best available evidence the standardised intervention is designed to be patient-centred, promote adherence to self-care, provide co-ordinated care including timely multidisciplinary team referrals, and increase access to supportive care for women who are sick or living in a remote setting, via the telephone. This novel program is innovative in several ways. First, it systematically engages peers over the treatment trajectory which is likely to reduce nursing time. Second, peers are linked in with the health professional team to encourage adherence to professionally-delivered information. Third, phone contact is adopted post-treatment completion to support those geographically or medically isolated. Finally, Level 1 evidence on preparing patients for threatening medical procedures is applied to the radiotherapy context.

We will employ a methodologically rigorous design, incorporating comprehensive selection, training, and monitoring of peers and nurses to ensure the intervention is feasible in a real-world clinical setting. Should this intervention be successful and widely disseminated, it has the potential to reduce the physical, psychosexual, and supportive care needs of women with GC. Additionally, meeting patients’ psychological and supportive care needs could have economic benefits as psychological morbidity can result in greater healthcare use [74]. In this new era which recognises consumers have an essential role in healthcare planning and delivery, the proposed research program has the potential to transform healthcare practices on an international level.

## Trial status

Patient recruitment is open.

## Competing interests

The authors declare that they have no competing interests.

## Authors’ contributions

PS and IJ conceptualised and designed the study. RB, KG, LM, MK, KW, DB, SP and SA assisted development of the protocol, study design and refinement of study materials. PS, IJ and KW will oversee implementation of the protocol and collection of data. PS, IJ and KW will provide health professional and peer volunteer training and supervision. PS led the writing of the protocol. All authors have been involved in drafting and critical evaluation of the manuscript. All authors have read and approved the final version.
